# Diffusion-negative MRI in acute ischemic stroke: a case report

**DOI:** 10.1186/1757-1626-1-65

**Published:** 2008-07-29

**Authors:** Rahul Rathakrishnan, Vijay K Sharma, Bernard PL Chan

**Affiliations:** 1 Division of Neurology, Department of Medicine, National University Hospital, Singapore

## Abstract

**Background:**

Diffusion-weighted magnetic resonance imaging is a very sensitive tool for the early diagnosis of acute ischemic stroke. This is employed in some stroke centers as the primary screening tool to select patients eligible for thrombolysis.

**Methods:**

We present the case of a 49-years old Chinese man whose diffusion-weighted magnetic resonance imaging performed 12 hours of symptom onset was negative.

**Results:**

Although the initial diffusion-weighted magnetic resonance imaging was negative, the imaging repeated after 4 days despite static neurological symptoms and signs, could demonstrate an acute medullary infarction.

**Conclusion:**

Diffusion-weighted imaging may not be100% sensitive in very early stages, especially in posterior circulation strokes. Our case serves as a reminder that clinical assessment still retains priority until a diagnostic modality offering 100% sensitivity and specificity is discovered.

## Introduction

Diffusion-weighted magnetic resonance imaging (DWI-MRI) is considered to be a very sensitive tool for the early diagnosis of acute ischemic stroke. This is employed in some stroke centers as the primary screening tool to select patients eligible for thrombolysis. However DWI may not be100% sensitive in very early stages, especially in posterior circulation strokes. We present a patient in which DWI-MRI was negative even after 12 hours of symptom onset and only the imaging repeated after 4 days could demonstrate an acute infarction.

## Case presentation

A 49-year old Chinese man, office clerk by occupation was admitted with sudden vertigo and unsteady gait. The vertigo was unremitting with eye closure and not posturally related. There was no trauma, neck pain or illness. There was no significant medical illness in past and accordingly, he was not on regular medication and did not smoke. Clinical examination revealed right lateral gaze-evoked nystagmus, right upper limb dysmetria and ataxic gait. Sensation to pain and temperature was intact and there was no evidence of a Horner's syndrome.

**Figure 1:**
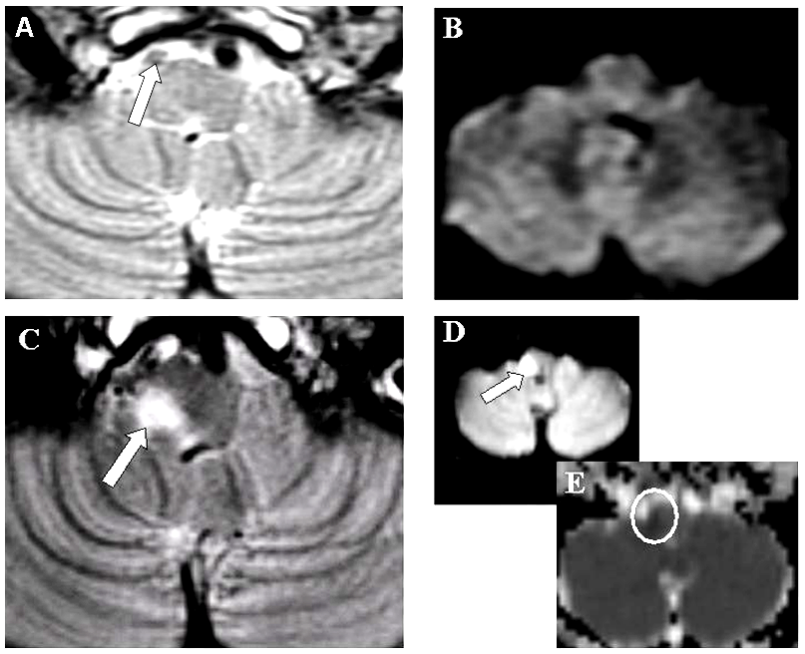
**Axial T2-weighted and diffusion weighted images**. **A and B**: Axial T2-weighted and diffusion weighted images (DWI) of the MRI 24 hours after presentation. The thin white arrow denotes the absent flow void of the right vertebral artery. **C, D and E**: Axial T2-weighted, DWI and apparent diffusion coefficient (ADC) of the same region 4 days later. The bold white arrows denote the infarct at the right medullary region and the white circle indicates the low ADC.

An emergent computerized tomography (CT) of the brain was unremarkable. An MRI of the brain including diffusion-weighted imaging (DWI) 24 hours after admission did not reveal an acute lesion. However, an absent right vertebral artery flow void on axial T2-weighted images was observed (Figure 1). Cervical duplex ultrasound of the extracranial vessels and transcranial Doppler were unremarkable for significant stenotic disease or arterial dissection. In view of the persistent neurological findings even after 4 days of the symptom-onset, an MRI of the brain was repeated that revealed a right medullary infarct on DWI (Figure 1). With continued therapy for secondary stroke prevention and intensive rehabilitative physiotherapy, he recovered completely in about 3 weeks.

## Discussion

Our patient demonstrates that DWI-MRI performed early may fail to demonstrate an acute infarct especially if it is small and located in territory supplied by posterior circulation. This may have an impact on the clinical decision making process in centres using DWI-MRI as a tool to guide aggressive therapy in patients with acute stroke [1]. Our case did not develop any new neurological signs and neuroimaging was repeated to ascertain if DWI would remain persistently negative despite static neurological deficits consistent with a cerebrovascular event.

In acute cell death, failure of sodium-potassium ATPase pumps result in the shift of water from extracellular to intracellular compartments, reflected as a hyperintense lesion on DWI [2]. DWI provides the earliest imaging evidence of ischemic injury [3] and can reveal abnormalities before other conventional MR sequences, even in transient ischemic events [4,5]. However DWI as an isolated sequence may fail to demonstrate an acute stroke in up to 20% cases [6]. Infarcts involving the medullary region yield highest false negative rates [7]. This becomes clinically relevant if MRI-DWI is employed as the imaging modality to determine attempts at recanalization during the acute treatment of ischemic stroke [8].

Technical reasons have been postulated for the occasional failure of DWI-MRI to demonstrate an acute infarction including poor spatial resolution, high signal-to-noise ratio and magnetic susceptibility artefacts around the brain stem [1]. Another important factor is the time elapsed from ictus at which the scan is performed. While positive DWI-MRI has been observed within 2.5 minutes of arterial occlusion in animal models, a longer duration (within 3 hours) in humans may not be sufficient for DWI changes despite a fixed neurological deficit [9,10]. Contrary to the previous reports of false-negative MRI-DWI within 3 hours of symptom-onset, our patient had a prolonged fixed neurological deficit for 24 hours and yet DWI failed to demonstrate an infarct despite flow-void abnormality in the right vertebral artery. We hypothesize that the point at which the reversible ischemia transforms into irreversible infarction and impaired diffusibility had not been crossed [7,9] when our patient had the initial MRI and thus the DWI remained negative. DWI abnormalities may be reflective of dynamic disturbances along different points of the ischemic cascade [3,7]. While 'restricted-diffusion' on DWI can occur with prolonged tissue hypoxia, the changes may be reversible if early reperfusion is achieved [3,7]. This indicates that MRI-DWI changes are not 'per se' absolute markers of ongoing or established ischemia and conversely, the *absence *of a DWI-lesion does not completely rule out an ischemic state.

This may be argued that the first MRI could have missed the particular section of the brain that underwent the ischemic infarct. This possibility, although very unlikely, can not be completely excluded. Another explanation for DWI-negative MRI could be an interval infarction between the two MRI scans performed in our case. However, our patient did not develop any new symptoms or signs suggestive of a new neurological injury.

In conclusion, if there is a high index of suspicion for an acute cerebrovascular event especially involving the posterior circulation with an unremarkable initial MRI-DWI, a repeat study after 24 hours of a fixed neurological deficit may be more demonstrative. Our case serves as a reminder that clinical assessment still retains priority until a diagnostic modality offering 100% sensitivity and specificity is discovered.

## Abbreviations

DWI: Diffusion-weighted imaging; MRI: Magnetic resonance imaging; CT: Computerized tomography.

## Competing interests

The authors declare that they have no competing interests.

## Authors' contributions

RR Patient management, initial draft of the manuscript, VKS Patient management, draft and review of manscript, conceptualization and final approval, BPLC Patient management, conceptualization, final approval.

## Consent

Written informed consent was obtained from the patient for publication of this case report and accompanying images. A copy of the written consent is available for review by the Editor-in-Chief of this journal.
